# Inguinal swelling unveiling biphasic synovial sarcoma: A case report

**DOI:** 10.1016/j.ijscr.2024.110630

**Published:** 2024-11-19

**Authors:** Faten Limaiem, Mohamed Hajri, Neirouz Kammoun, Taher Laabidi, Zied Hadrich, Nidhameddine Kchir

**Affiliations:** a University of Tunis El Manar, Faculty of Medicine of Tunis, 1007, Tunisia; b Department of Pathology, Hospital Mongi Slim La Marsa, Tunisia; c Department of Surgery, Hospital Mongi Slim La Marsa, Tunisia; d Private Pathology Laboratory, Tunis, Tunisia

**Keywords:** Synovial sarcoma, Soft tissue sarcoma, Inguinal tumor, Surgery, Pathology, Immunohistochemistry

## Abstract

**Introduction and importance:**

Synovial sarcoma is a high-grade soft tissue sarcoma primarily affecting teenagers and young adults. Inguinal region involvement is rare, making diagnosis and treatment challenging.

**Case presentation:**

A 35-year-old Tunisian man presented with a progressively enlarging right inguinal swelling. Imaging revealed a mass behind the inguinal ligament, confirmed as biphasic synovial sarcoma through biopsy. The patient underwent successful surgery with clear resection margins. Histopathological examination revealed a biphasic sarcoma with spindle cell and glandular components, supporting the diagnosis of synovial sarcoma. Following surgery, the patient received adjuvant radiotherapy. Regular outpatient follow-up is being conducted to monitor progress.

**Clinical discussion:**

Synovial sarcoma is characterized by slow growth and local invasiveness, with potential for metastasis. It typically presents as a solid mass that can compress nearby structures such as blood vessels. Imaging studies offer valuable insights into tumor location, size, invasiveness, and potential metastases. Local tumor staging relies on MRI, while distant metastases are detected using chest CT or bone scans. Diagnosis is confirmed through histopathological examination and immunohistochemical analysis.

**Conclusions:**

This case report highlights a rare presentation of inguinal synovial sarcoma and emphasizes the importance of individualized multimodal therapy in its management.

## Highlights

• Inguinal synovial sarcoma is rare, posing diagnostic challenges.
• The initial signs of inguinal synovial sarcoma can mimic other lesions, complicating diagnosis.
• Clear resection margins are crucial in surgically managing large inguinal synovial sarcoma for successful treatment.
• Individualized therapy is key for inguinal synovial sarcoma due to the lack of a standard treatment protocol.
• Regular follow-up is vital for monitoring progress and detecting potential recurrence.
• This case report provides valuable insights into managing rare synovial sarcoma presentations, addressing literature gaps.

## 1 Introduction

Synovial sarcoma (SS) is a high-grade soft tissue sarcoma that most commonly arises near large joints in the extremities. It primarily affects adolescents and young adults and is characterized by a chromosomal translocation that leads to the formation of the SYT-SSX fusion protein [1,2]. Although SS has been reported in a variety of atypical locations, primary SS in the inguinal region is exceedingly rare, with only six cases documented in the English-language literature to date [1,2]. Diagnosing inguinal SS can be challenging due to its nonspecific initial signs and symptoms, which often resemble other lesions in the region. Due to the aggressive nature of SS, comprehensive pathological and radiological evaluations are essential for staging and determining the extent of the lesion to inform appropriate treatment. While imaging studies often offer important diagnostic insights, an accurate diagnosis of SS requires histopathological examination coupled with immunohistochemical analysis. In this paper, the authors present a unique case of a large inguinal SS and discuss updated multimodal treatment strategies for soft tissue sarcomas, aiming to improve the understanding and management of this rare presentation. This case report adheres to the SCARE Criteria [3].

## 2 Case presentation

**Figs. 1 and 2:**
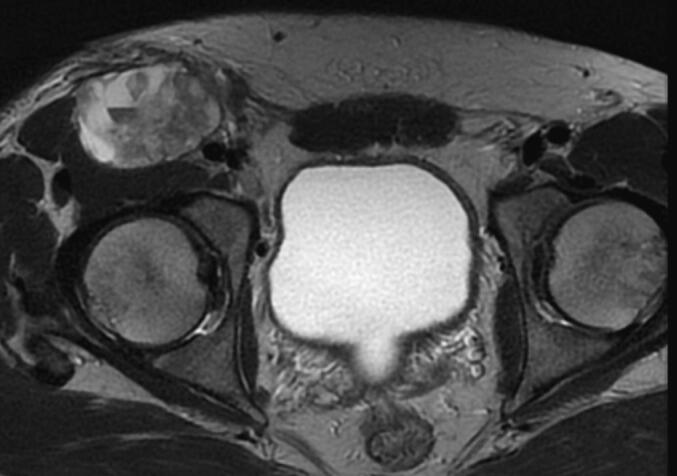

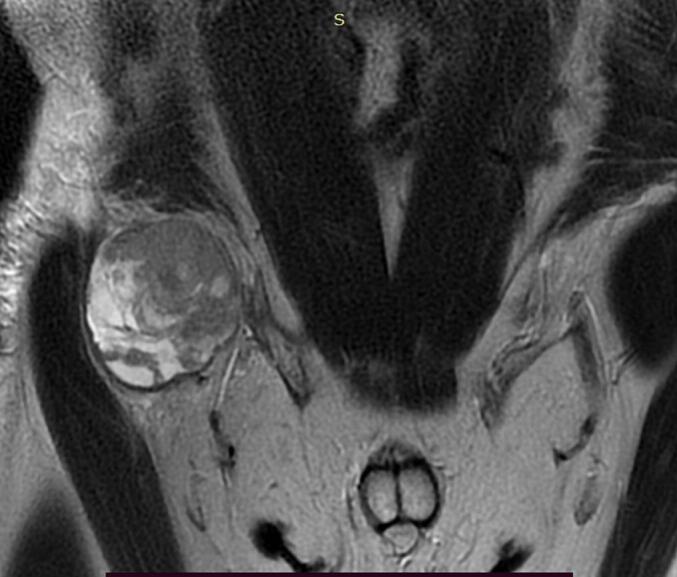
Pelvic MRI displaying a hyperintense mass on T1-weighted imaging developed in the right inguinal region behind the inguinal ligament and the inguinal fascia. It measures 40 mm in antero-posterior diameter, 60 mm in height, and 62 mm in transverse diameter.

**Fig. 3:**
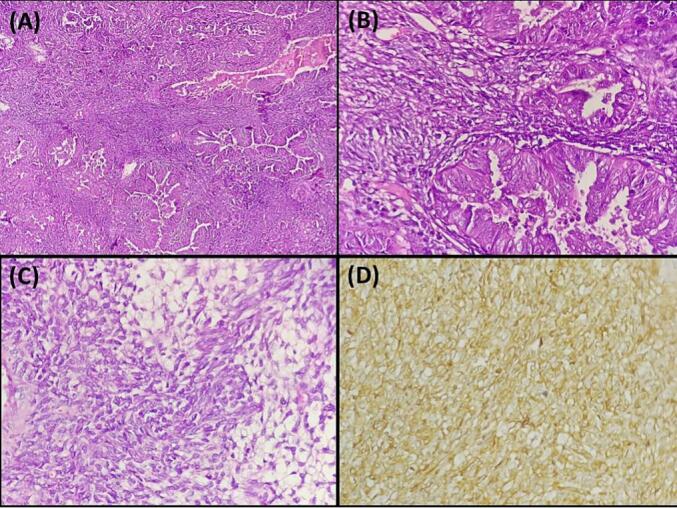
A: Histopathological image illustrating a biphasic synovial sarcoma: The image depicts a distinct biphasic pattern characterized by the coexistence of mesenchymal and epithelial differentiation within the tumor (hematoxylin and eosin, magnification × 100). B: Biphasic synovial sarcoma: Image shows spindle cell areas with synovial sarcoma nuclear features and closely packed glandular cells (hematoxylin and eosin, magnification × 400). C: Histopathological image of biphasic synovial sarcoma: The image depicts the mesenchymal component of the tumor, characterized by focal myxoid changes (hematoxylin and eosin, magnification × 400). D: Immunohistochemical staining of synovial sarcoma showing CD99 positivity: The image demonstrates brown staining, indicating positive expression of CD99 in the tumor (immunohistochemistry, × 400).

A 35-year-old Tunisian man with an unremarkable medical history presented with pain and a progressively enlarging right inguinal swelling over the past 6 months. On examination, a hard and tender mass measuring 6 cm was palpable in the right inguinal region, without signs of local inflammation. The mass was fixed in relation to the deep plane, and there was no sensory or motor deficit in the right lower limb. An inguinal ultrasound revealed a solid-cystic mass measuring 60 mm. Subsequent pelvic MRI identified a mass located in the right inguinal region behind the inguinal ligament and fascia, measuring 40 mm in antero-posterior diameter, 60 mm in height, and 62 mm in transverse diameter (Figs. 1 and 2). While no distant metastases were found, the mass was adherent to the iliac muscle with a compromised safety margin, extending above the inguinal ligament and in contact with the common femoral artery without invasion, suggestive of locally advanced synovial sarcoma. An MRI-guided core biopsy was performed and histological examination established that the mass was sarcomatous. The patient underwent surgery with wide excision of the mass into healthy tissue. Histopathological examination revealed a biphasic malignant tumor proliferation consisting of spindle cell and glandular components (Fig. 3A, B). The spindle cell component displayed elongated cells in interlacing fascicles or whorls with focal myxoid changes (Fig. 3C), showing mild nuclear atypia with elongated nuclei, vesicular chromatin, and prominent nucleoli. The glandular component comprised closely packed round to ovoid cells forming gland-like structures, some containing intraluminal eosinophilic material and lined by cuboidal to columnar epithelial cells (Fig. 3B). The mitotic index was 11 mitoses per 10 high power fields, with focal areas of necrosis <50 %. Immunohistochemically, the spindle cell component was positive for vimentin, CD99 (Fig. 3D), and BCL2, while the glandular component expressed epithelial markers such as cytokeratin CK7 and EMA. The final pathological diagnosis was Grade 3 biphasic synovial sarcoma, with clear resection margins indicating complete tumor removal. The postoperative course was uncomplicated, and the patient was subsequently referred for adjuvant radiotherapy to optimize treatment outcomes. Currently, the patient is undergoing regular outpatient follow-up to monitor their progress and ensure appropriate surveillance.

## 3 Discussion

SS is a rare and aggressive type of soft tissue cancer, representing approximately 8 to 10 % of all soft tissue sarcomas [1]. It typically develops near the large joints in the limbs. The most common primary sites for SS are the extremities (68.7 %) and trunk (15.7 %) [4,5]. However, its occurrence in the inguinal region is extremely rare, with only six reported cases documented in the literature to date (Table 1) [[4], [5], [6], [7]]. SS primarily affects young adults, particularly those in their second and third decades of life, with a slightly higher incidence in males [1,8]. The cellular origin of SS involves the dysregulation of self-renewal in disordered mesenchymal stem cells, mediated by the SS18-SSX fusion protein [9]. This fusion protein plays a significant role in the pathogenesis of SS. Patients with SS commonly exhibit nonspecific symptoms such as swelling or pain resulting from the compression of neighboring tissues. Additionally, individuals with SS may experience persistent pain at the tumor site before noticeable swelling occurs [1]. Our patient presented with pain and a progressively enlarging right inguinal swelling over six months. In most cases, patients are diagnosed with localized disease, although approximately 6 % to 18 % present with metastatic disease at the time of diagnosis [1,7]. When evaluating inguinal masses, a comprehensive differential diagnosis spans various categories, encompassing congenital anomalies, non-congenital hernias, vascular disorders, inflammatory or infectious conditions, and neoplasms. This diagnostic spectrum includes diverse conditions such as inguinal and femoral hernias, reactive or malignant adenopathy, iliac or femoral aneurysms, sebaceous cysts, epididymitis, testicular torsion, lipomas, hidradenitis, varicoceles, hydroceles, ectopic or undescended testicles [10]. Imaging studies play a crucial role in diagnosing SS by providing valuable insights into tumor location, size, invasiveness, and potential metastases [11]. Although the radiographic features of these tumors are not pathognomonic, the presence of a soft-tissue mass, particularly if calcified (30 %), near but not within a joint in a young patient, strongly suggests the diagnosis [11]. MRI is considered the gold standard for local staging of the tumor. It provides detailed information about the size, location, and extent of the tumor within the soft tissues [11]. SS may appear as small, homogenous nodules with minor mass effects or as large, heterogeneous masses encompassing vessels and nerves. Some SS demonstrate benign imaging characteristics, with lesion compositions varying from pure cystic to mixed solid/cystic. Hemorrhagic and calcific foci are often visible. Certain signal patterns on T2-weighted imaging can signify large masses [11]. The typical CT appearance of SS is a heterogeneous deep-seated soft-tissue mass with attenuation similar to or slightly lower than that of muscle. It may exhibit areas of lower attenuation suggestive of necrosis or hemorrhage, although smaller lesions can appear more homogeneous. CT scans are valuable for identifying calcifications and bone involvement in SS, especially in intricate anatomical regions like the pelvis, hip, or shoulder, or when lesions are small and subtle [11]. The diagnosis of SS is based on histopathological examination along with an immunohistochemical study. Grossly, SS typically presents as well-defined nodules, ranging from 3 to 10 cm at diagnosis, with smaller lesions (<1 cm) also observed, especially in the hands and feet. The tumor's cut surface may vary in color (tan, grey, yellow, or pink) and texture (soft or firm) [12]. Histologically, SS is categorized into monophasic, biphasic, and poorly differentiated variants [12]. In our patient, the biphasic subtype was identified, characterized by spindle cells and gland-like epithelial structures. Monophasic SS is distinguished by infiltrative borders and a hypercellular fascicular architecture, while poorly differentiated SS consists of highly cellular round cells. Additional features of SS include focal staghorn branching vascular patterns, the common presence of mast cells, and occasional focal calcification. Positive stains in SS include TLE1, Cytokeratins, EMA, BCL2, beta-catenin, calponin, CD99, CD56, CD57, calretinin, as well as specific antibodies for SS18-SSX fusion and SSX C terminus [12]. For localized SS, the standard treatment approach involves surgical resection with wide excision aiming for negative margins whenever feasible, coupled with radiotherapy (neoadjuvant or adjuvant) for intermediate-high-grade (grades 2–3), deep-seated, and lesions larger than 5 cm. The utility of neoadjuvant chemotherapy in SS patients is still a subject of uncertainty, particularly in terms of patient selection for potential benefits and determining the optimal duration of treatment cycles [13]. While ESMO guidelines suggest three cycles of neoadjuvant full-dose anthracycline plus ifosfamide chemotherapy over five cycles due to comparable overall survival rates, treatment decisions for rarer SS histological subtypes should be personalized [13]. In advanced cases of SS, anthracycline-based chemotherapy is the standard first-line treatment. The prognosis of SS varies, with 5-year survival rates ranging from 36 % to 76 % [14]. A study has identified several factors that contribute to longer survival, including female gender, belonging to a nonblack race, having an extremity tumor, a localized tumor, and a tumor size smaller than 5 cm. It is worth noting that the most common sites of metastasis for synovial sarcoma are the lungs and bones [14].

**Table 1:** Details of primary inguinal synovial sarcoma in literature review.

Study	Year	Age/sex	Management	Diagnosis	Follow-up
Sohrabi et al. [3]	1988	47/M	Surgical excision	SS in groin	No evidence of recurrence after 56 months follow-up
van der Heidi [4]	2000	19/F	Neoadjuvant chemoradiotherapy + surgical excision	Biphasic SS from femoral neck	No evidence of recurrence after 24 months of follow-up
Naito [5]	2010	68/F	Surgical excision	Monophasic SS in groin	Lung and spleen metastases were observed 15 months after surgery, and patient died 24 months after surgery
Xu [6]	2015	53/M	Surgical excision + adjuvant chemoradiotherapy	Monophasic SS of spermatic cord	No evidence of recurrence after 36 months of follow-up
Chen WC et al. [2]	2020	72/M	Surgical excision Patient refused adjuvant chemoradiotherapy	Biphasic SS	No evidence of recurrence after 12 months of follow-up
Chaker J et al. [1]	2024	23/M	Surgical excision + adjuvant chemoradiotherapy	Biphasic SS	No evidence of recurrence after 24 months of follow-up
Present case	2024	35/M	Surgical excision + adjuvant radiotherapy	Biphasic SS	No evidence of recurrence after 18 months of follow-up

This case report highlights the unusual occurrence of SS in the inguinal region, a rare presentation of this tumor. The report's strength lies in its thorough diagnostic approach, integrating various imaging and biopsy techniques to delve into the molecular complexities of this neoplasm. Despite the detailed analysis, limitations such as a small sample size and the lack of comparative data underscore the necessity for further research to establish standardized management practices for this unique clinical entity. In essence, inguinal SS, a rare high-grade soft tissue sarcoma, poses diagnostic challenges due to its vague initial symptoms. Early recognition and precise diagnosis are crucial for guiding appropriate treatment, which typically involves multidisciplinary approaches such as surgical excision and adjuvant therapy. Collaborative efforts are essential to formulate standardized management protocols for this uncommon condition.

## Consent

Written informed consent was obtained from the patient for publication of this case report and accompanying images. A copy of the written consent is available for review by the Editor-in-Chief of this journal on request.

## Provenance and peer review

Not commissioned, externally peer-reviewed.

## Ethical approval

Ethical approval for this study was provided by the Ethical Committee of Mongi Slim University Hospital, Marsa, Tunisia.

## Funding

This research did not receive any specific grant from funding agencies in the public, commercial, or not-for-profit sectors.

## Guarantor

Dr. Faten Limaiem.

## CRediT authorship contribution statement

Dr. Faten Limaiem and Dr. Mohamed HAJRI: Prepared, organized, wrote, and edited all aspects of the manuscript.
Dr. Neirouz Kammoun, Dr. Taher Laabidi, Dr. Zied Hadrich, Pr Nidhameddine Kchir: Read, edited, and approved the final version of the manuscript. Contributed to data acquisition, analysis, and interpretation. Provided final approval of the manuscript before its submission.

## Declaration of competing interest

None declared.
